# Supplementary material for: A fault-intrusion-tolerant system and deadline-aware algorithm for scheduling scientific workflow in the cloud
Source: PeerJ Comput Sci. 2021 Nov 2;7:e747. doi: 10.7717/peerj-cs.747 (PMC8576554; doi:10.7717/peerj-cs.747)
Supplement: Supplemental Information 6 [file peerj-cs-07-747-s006.zip › Myproject1.1/docs/org/cloudbus/cloudsim/power/models/class-use/PowerModelSpecPower.html]

Uses of Class org.cloudbus.cloudsim.power.models.PowerModelSpecPower


JavaScript is disabled on your browser.


- Overview
- Package
- Class
- Use
- Tree
- Deprecated
- Index
- Help

- Prev
- Next

- Frames
- No Frames

- All Classes

## Uses of Class org.cloudbus.cloudsim.power.models.PowerModelSpecPower

- Packages that use PowerModelSpecPower

  | Package | Description |
  | --- | --- |
  |  |  |
  | --- | --- |
  | org.cloudbus.cloudsim.power.models |  |
- - ### Uses of PowerModelSpecPower in org.cloudbus.cloudsim.power.models

    Subclasses of PowerModelSpecPower in org.cloudbus.cloudsim.power.models

    | Modifier and Type | Class and Description |
    | --- | --- |
    |  |  |
    | --- | --- |
    | `class` | `PowerModelSpecPowerHpProLiantMl110G3PentiumD930` The power model of an HP ProLiant ML110 G3 (1 x [Pentium D930 3000 MHz, 2 cores], 4GB). |
    | `class` | `PowerModelSpecPowerHpProLiantMl110G4Xeon3040` The power model of an HP ProLiant ML110 G4 (1 x [Xeon 3040 1860 MHz, 2 cores], 4GB). |
    | `class` | `PowerModelSpecPowerHpProLiantMl110G5Xeon3075` The power model of an HP ProLiant ML110 G5 (1 x [Xeon 3075 2660 MHz, 2 cores], 4GB). |
    | `class` | `PowerModelSpecPowerIbmX3250XeonX3470` The power model of an IBM server x3250 (1 x [Xeon X3470 2933 MHz, 4 cores], 8GB). |
    | `class` | `PowerModelSpecPowerIbmX3250XeonX3480` The power model of an IBM server x3250 (1 x [Xeon X3480 3067 MHz, 4 cores], 8GB). |
    | `class` | `PowerModelSpecPowerIbmX3550XeonX5670` The power model of an IBM server x3550 (2 x [Xeon X5670 2933 MHz, 6 cores], 12GB). |
    | `class` | `PowerModelSpecPowerIbmX3550XeonX5675` The power model of an IBM server x3550 (2 x [Xeon X5675 3067 MHz, 6 cores], 16GB). |

- Overview
- Package
- Class
- Use
- Tree
- Deprecated
- Index
- Help

- Prev
- Next

- Frames
- No Frames

- All Classes
